# Risk factors for neck pain in office workers: a prospective study

**DOI:** 10.1186/1471-2474-7-81

**Published:** 2006-10-25

**Authors:** Julia M Hush, Chris G Maher, Kathryn M Refshauge

**Affiliations:** 1 School of Physiotherapy, Faculty of Health Sciences, University of Sydney, PO Box 170 Lidcombe, NSW 1825, Australia

## Abstract

**Background:**

Persisting neck pain is common in society. It has been reported that the prevalence of neck pain in office workers is much higher than in the general population. The costs to the worker, employer and society associated with work-related neck pain are known to be considerable and are escalating. The factors that place office workers at greater risk of developing neck pain are not understood. The aim of this study is to investigate the incidence and risk factors of work-related neck pain in Australian office workers.

**Methods/design:**

We will conduct a prospective cohort study. A cohort of office workers without neck pain will be followed over a 12 month period, after baseline measurement of potential risk factors. The categories of risk factors being evaluated are physical (cervical spine posture, range of movement, muscle endurance and exercise frequency), demographic (age, sex), work environment (sitting duration, frequency of breaks) and psychosocial (psychological distress and psychosocial work factors). Cox regression analysis will be used to identify risk factors associated with work-related neck pain, and will be expressed as hazard ratios with 95% confidence intervals. The data will also enable the incidence of neck pain in this population to be estimated.

**Discussion:**

In addition to clarifying the magnitude of this occupational health problem these data could inform policy in workplaces and provide the basis for primary prevention of neck pain in office workers, targeting the identified risk factors.

## Background

Normal neck function underpins successful performance of activities of daily living. In the general population, neck pain and dysfunction are common, affecting up to 67% of the general population at some time during their life [1]. The one year prevalence of neck pain has been reported as 32% in a population of Hong Kong Chinese [2]. Neck pain may arise from any of the innervated structures in the neck, such as intervertebral discs, muscles, ligaments, zygapophyseal joints, dura or nerve roots [3]. However in the majority of cases, the pathophysiological mechanisms underlying neck pain are unclear. Such "non-specific" neck problems are costly in terms of disability and work loss [4]. Estimates indicate that the economic consequences of treating disabling chronic neck pain are significant. For instance in the Netherlands annual costs associated with chronic non-specific neck pain have been reported at US$868 million [5].

Many studies have investigated the relationship between neck pain and working conditions. Previous research has identified that office workers are a specific population at high risk of developing neck pain, with one year prevalence rates much higher than in the general population [6-8]. For example, one year prevalence of neck pain in office workers at a Hong Kong university was found to be 59% [6] and 63% in a Swedish study of medical secretaries [7]. While neck pain is generally believed to be of multifactorial origin [9], it remains unclear which factors place office workers, in particular, at higher risk. Postulated factors in this occupational group include: individual factors (e.g. sex) [7,10,11], work environment factors (e.g. repetitive work, exposure level) [7,11,12] psychosocial factors (e.g. stress, high job demands, low decision latitude) [11,12] and perceived muscular tension [11,12]. However, methodological considerations limit interpretation of these studies either because of high loss to follow-up (22% [11] to 48% [12]) in the longitudinal studies, which can introduce significant bias into the study findings, or cross-sectional design.

Physical risk factors (such as prolonged sitting and neck flexion) have been identified as predictive of neck pain in the study of a mixed population of workers from various industry, health and professional settings [13]. These and other physical factors (such as posture and neck muscle endurance) have not been prospectively investigated specifically in office workers. Physical risk factors are useful to investigate as they are potentially reversible with exercise-based intervention[14]. It has been argued that both physical and psychosocial contributors to work-related neck pain need to be assessed together in longitudinal designs [15], to evaluate their relative contribution to the onset of work-related musculoskeletal pain. Such longitudinal studies are lacking, especially in a population of office workers. The incidence and risk factors for work-related neck pain in Australian office workers remain unknown.

The aims of this project are to determine factors that predict neck pain in Australian office workers and to attain an estimate of the incidence of neck pain in this population. Individual, workplace, psychosocial and physical factors will be investigated as potential predictors in this exploratory longitudinal study. It is anticipated that this project will provide data to inform the design of a larger definitive study.

## Methods/design

### Design

**Figure 1:**
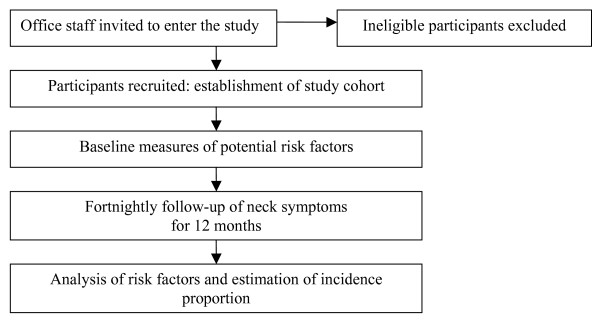
Study flow chart.

A prospective cohort study will be conducted to determine factors that increase the risk of development of neck pain in office workers. A cohort of office workers without neck pain will be assessed at baseline and then prospectively followed over a 12 month period. Data will be analysed to estimate the incidence of neck pain in this population sample and to identify risk factors. The study co-ordinator will be blinded to the baseline measures to minimise bias during follow-up and data analysis. A flow chart of the study process is illustrated in Figure 1. The study procedures and measures have been approved by the University of Sydney Human Research Ethics Committee.

### Recruitment

A random sample of 100 study participants will be drawn from a population of office workers at tertiary education institutions, inviting both academic and general staff and research students to participate. This sample of convenience was chosen because university staff have been shown to have higher prevalence of neck pain than the general community[6]. Sampling will be achieved by using a randomisation schedule which will be computer generated.

Letters inviting office workers to participate in the study will be mailed out with participant information sheets. A trained health researcher will screen volunteers to confirm eligibility by telephone interview. The first 100 eligible volunteers will form the study cohort.

### Eligibility

Participants will be included if they are employed full-time, speak and read English and are aged between 18 and 60 years. Participants will be excluded if they have neck pain currently, any specific medical condition affecting the cervical spine (such as ankylosing spondylitis, tumour, infection, rheumatoid arthritis), a prolonged absence from work anticipated within the next 12 months or a history of an episode of care for neck pain in the past three months. An episode of care for neck pain is defined as *a consultation or series of consultations with a health professional, for neck pain*. This definition has been modified for neck pain from the definition of an episode of back pain [16]. The standardized Nordic Questionnaire definition of neck pain will be employed in the study, supplemented by a pain-region drawing [17].

### Sample size

As an exploratory study, a sample size of 100 was determined to be sufficient to provide information for the larger definitive study.

### Baseline measures

Baseline measures of putative risk factors will be assessed using both self-report questionnaires and measures of physical neck function (Table 1).

#### Physical factors

Neck range of movement and endurance capacity will be measured. Neck range of movement (flexion, extension, lateral flexion and rotation) will be measured using the Cervical Range of Movement Instrument[18]. Cervical spine posture (protraction) in usual sitting will be measured using the same instrument. Endurance of the cervical extensor muscles will be measured using the Cervical Biering-Sorenson Test[19]. Self-reported estimates of frequency of exercise will be collected by questionnaire.

#### Individual demographic characteristics

Age and gender will be collected by questionnaire.

#### Work environment factors

Total duration of daily sitting at work and frequency of breaks from sitting will be evaluated by self-reported estimates.

#### Psychosocial factors

Psychosocial workplace factors will be assessed using the Job Content Questionnaire(JCQ) [20]. This instrument is a widely-used measure of psychosocial stress at work comprising 3 domains: mental work-load (psychological job demands), decision latitude and social support. The standard version of the JCQ will be used, consisting of 49 questions, supplemented with the job dissatisfaction subscale. General psychological distress will be assessed using the overall score on the DASS21 instrument, a self-report measure that evaluates depression, anxiety and stress [21].

### Subject follow-up

Participants will be followed-up during work hours once per fortnight, by email or telephone, according to preference. The primary question to be asked at each follow-up is: "*Have you experienced any neck pain lasting more than 24 hours during the past fortnight *(Y/N)?"

If a participant reports any onset of neck pain, information will be obtained regarding date of onset, treatment sought and work loss. Additionally, the participant will be questioned to ascertain whether the episode is work-related. A positive response to any of the following questions confirms that the neck pain is work-related:

1. Did your neck pain start at work?

2. Did your neck pain result from an injury or event at work?

3. Have you submitted a worker's compensation claim for this episode of neck pain?

4. Did your health care provider specify that this is a work-related injury?

In the event of a participant reporting an episode of neck pain, he/she will continue to be followed up for a further period of one month to establish the type of neck pain according to the definitions listed in Table 2 (modified from definitions of episodes of back pain [15].

If a subject reports an episode of neck pain that is deemed not to be work-related (eg. following an injury occurring outside of work hours), the subject will be withdrawn from the study. If appropriate, general advice to consult a health care practitioner may be provided.

### Data analysis

Risk factors for work-related neck pain will be analysed using Cox regression analysis, and will be expressed as hazard ratios with 95% confidence intervals. Factors will be considered significant at a level of p < 0.05. Separate primary analyses will be conducted for each of three types of neck pain episodes (as defined in Table 2). An estimate of incidence proportion will be calculated as the proportion of new cases of work-related neck pain in the cohort during the 12 month period of observation. Statistical analysis will be conducted using the SPSS 14.0 software program (SPSS Inc., Chicago, IL).

## Discussion

This protocol describes a prospective cohort study that aims to determine the incidence of neck pain in Australian office workers and to identify risk factors associated with the onset of work-related neck pain. The results of this study will be useful to inform aspects of design for a larger definitive study, such as determination of sample size, feasibility and recruitment, and the workability of definitions of work-related neck pain. In the longer term, determination of factors that predict neck pain in office workers will be critical in the development of preventative workplace interventions and potentially occupational health and safety policy.

## Competing interests

The author(s) declare that they have no competing interests.

## Authors' contributions

JH conceived of the study and participated in its design as well as drafting the manuscript. CM and KR participated in the design of the study and contributed to writing the manuscript. All authors read and approved the final manuscript.

## Pre-publication history

The pre-publication history for this paper can be accessed here:



## Figures and Tables

**Table 1:** Risk factors of work-related neck pain being evaluated.

**Risk Factor**	**Construct**	**Measurement method**
Active range of cervical rotation	Total excursion of rotation (measured as the sum of right and left rotation), in degrees.	Cervical Range of Movement Instrument (CROM)
Active range of cervical lateral flexion	Total excursion of lateral flexion (measured as the sum of right and left lateral flexion), in degrees.	CROM
Active range of cervical flexion and extension	Total excursion of flexion and extension (measured as the sum of flexion and extension), in degrees.	CROM
Neck posture	Cervical protraction in relaxed sitting position (horizontal distance in cm)	CROM
Neck extensor endurance	Time that subject can maintain horizontal head position in prone (holding time in seconds)	Cervical Biering-Sorensen Test
Age	Age (years)	Questionnaire
Sex	M or F	Questionnaire
Psychological distress	Total score (/63)	DASS21
Psychosocial workplace factors	Total score (/264)	Job Content Questionnaire
Duration of daily sitting at work	Self-reported average duration of total sitting at work (hours)	Questionnaire
Frequency of breaks from sitting at work	Self-reported average frequency of breaks from sitting at work (minutes)	Questionnaire
Exercise frequency	Self-reported average frequency of weekly exercise sessions (sessions/week)	Questionnaire

**Table 2:** Definitions of episodes of neck pain to be employed in the study.

**Type of episode**		**Definition**
**Type 1**	Episode of neck pain	A period of neck pain lasting for more than 24 hours.
**Type 2**	Episode of care for neck pain	A period of neck pain lasting for more than 24 hours, with consultation or a series of consultations to a health professional.
**Type 3**	Episode of work absence due to neck pain	A period of neck pain lasting for more than 24 hours and a period of work absence due to neck pain.
